# Disseminated Kaposi Sarcoma in a Patient With Hematemesis: A Case Report

**DOI:** 10.7759/cureus.72262

**Published:** 2024-10-24

**Authors:** Curry Sherard, Samantha Parks, Halle Field

**Affiliations:** 1 Department of Internal Medicine, Medical University of South Carolina, Charleston, USA

**Keywords:** chemotherapy, disseminated kaposi sarcoma, gastrointestinal kaposi's sarcoma, hiv aids, national institutes of health, pulmonary kaposi sarcoma, visceral kaposi sarcoma

## Abstract

Kaposi sarcoma (KS) is a soft tissue tumor primarily occurring in immunosuppressed patients and, although it has been described, dissemination of KS is rare. This case involves a patient with acquired immunodeficiency syndrome (AIDS) and biopsy-proven cutaneous KS presenting with hematemesis suspected due to gastrointestinal involvement in whom endoscopy with biopsy was contraindicated due to dual antiplatelet therapy (DAPT) and the hypervascular nature of KS tumors. Alternatively, the diagnosis was made via macroscopic findings on bronchoscopy, which demonstrated neovascular lesions, confirming visceral KS. Treatment of disseminated KS generally consists of a combination of high-activity antiretroviral therapy (HAART) and chemotherapy; however, due to financial concerns, this patient was transferred to the National Institutes of Health (NIH), where he received free systemic chemotherapy. On follow-up less than three months later, he had no further symptoms or pulmonary lesions. This case adds to current knowledge by providing alternative pathways for diagnosis of and treatment strategies for patients with disseminated Kaposi Sarcoma.

## Introduction

Kaposi sarcoma (KS) is a mucocutaneous vascular tumor caused by Kaposi sarcoma-associated herpesvirus/human herpesvirus 8 (KSHV/HHV-8) [1]. HHV-8 infection in the setting of immune dysfunction leads to overexpression of lymphatic-associated genes in affected cells causing uncontrolled proliferation and the characteristic vascular tumors. This disease is well known for its cutaneous manifestations; however, rarely it will disseminate to visceral mucosa in the severely immunocompromised [1]. Visceral involvement, particularly in the lungs, is a negative prognostic factor, and therefore, it is imperative that clinicians have a strategy for recognizing, diagnosing, and treating this condition [1]. There are four clinical forms of KS: classic, African (endemic), AIDS-associated, and iatrogenic [1-3]. Of these forms, AIDS-associated and iatrogenic have been associated most with visceral involvement [1]. AIDS-KS is the second most common tumor in HIV patients with CD4 counts < 200 cells/mm3, with an incidence of up to 30%. HIV-positive male homosexuals have a five- to ten-fold increased risk of KS [1].

## Case presentation

**Figure 1:**
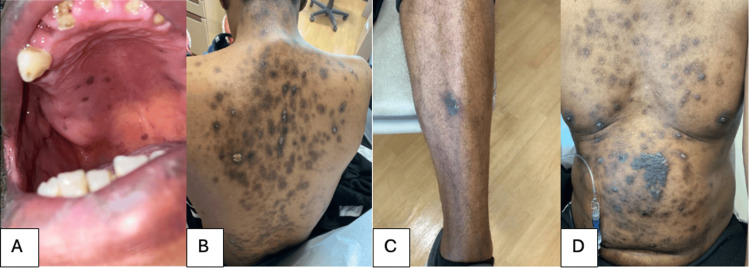
Vascular lesions on the patient’s oral mucosa (A), back (B), extremities (C), and trunk (D).

The patient is a 51-year-old male with HIV/AIDS and not on highly active antiretroviral therapy (HAART), with a recent neurosyphilis infection, with right pontine stroke on dual antiplatelet therapy (DAPT), and with biopsy-proven cutaneous KS. He presented to the clinic and was noted to have active hematemesis, at which point, he was transferred to the emergency department. The patient endorsed a three-day history of multiple episodes of bright red hematemesis daily. He otherwise denied nausea, diarrhea, constipation, or abdominal pain. He was previously on HAART before becoming lost to follow-up over 10 years prior. He was tachycardic with his heart's beats per minute in the 150s, but all other vital signs were stable. On skin examination, he had numerous purple-colored patches and plaques over his extremities, trunk, back, and oral mucosa as shown in Figure 1.

**Figure 2:**
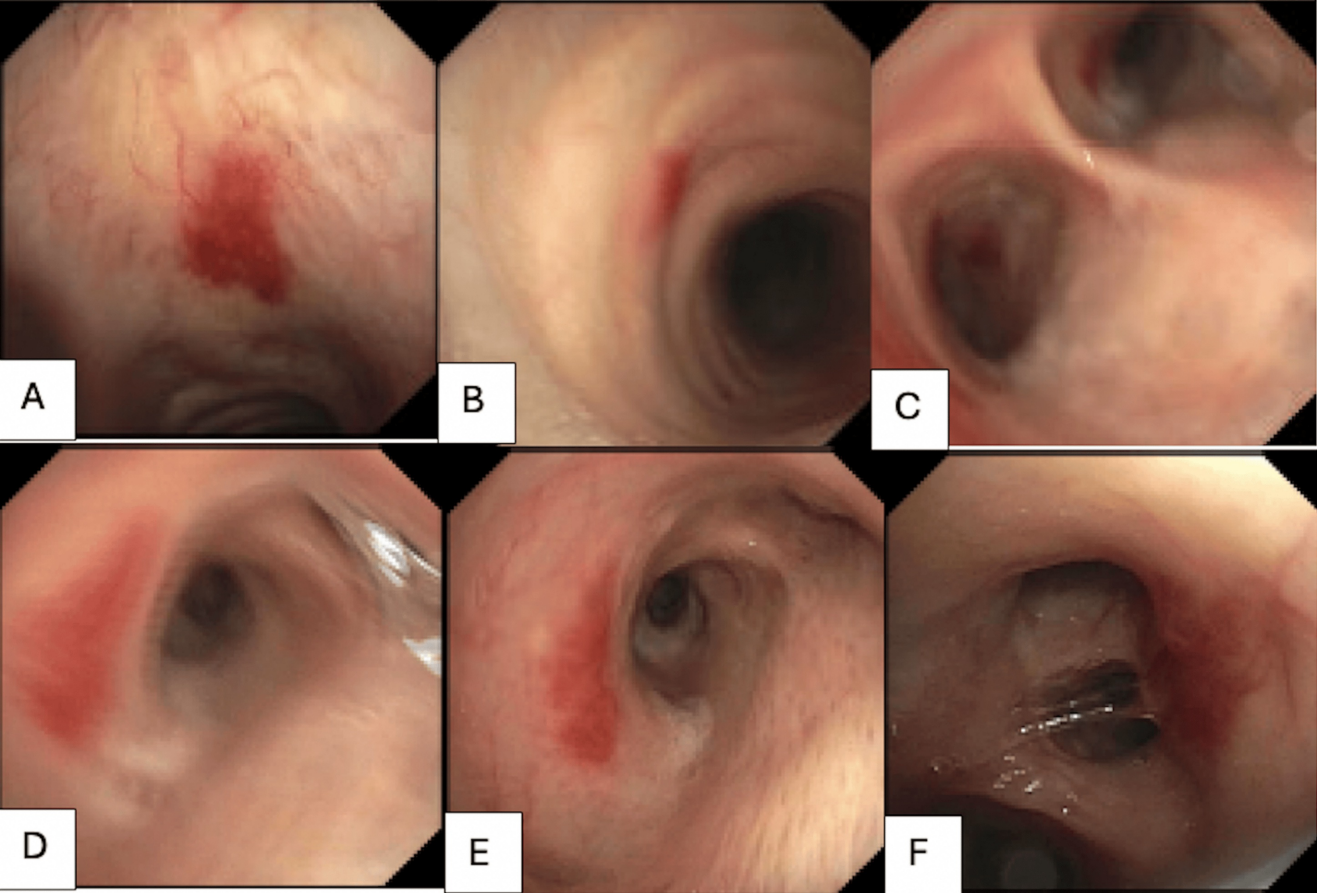
Neovascular lesions visualized during bronchoscopy in different locations of the tracheobronchial tree including the trachea (A, B), carina (C), right mainstem bronchus (D), and left mainstem bronchus (E, F).

His hemoglobin on admission was 10.2 gm/dL (reference range 14.0-18.0 gm/dL) and dropped to 7.7 gm/dL within 24 hours. His most recent HIV-1 RNA viral load was 44,769 copies/mL (reference range not detected copies/mL) with CD4 count <35 uL (reference range 362-1531 uL) and an HHV-8 count of 600 copies/mL (reference range not detected copies/mL). He received IV fluids, packed red blood cells, trimethoprim-sulfamethoxazole for pneumocystis jirovecii prophylaxis, an IV proton pump inhibitor, and was placed on a clear liquid diet for possible endoscopy. Gastroenterology, pulmonology, and infectious disease (ID) specialists were consulted. Ultimately, endoscopy was deemed to be contraindicated due to recent DAPT use. In the setting of high suspicion for visceral spread of KS, the pulmonologist recommended bronchoscopy to evaluate further. Neovascular lesions were visualized throughout the tracheobronchial tree as shown in Figure 2 and confirmed a diagnosis of visceral KS.

**Figure 3:**
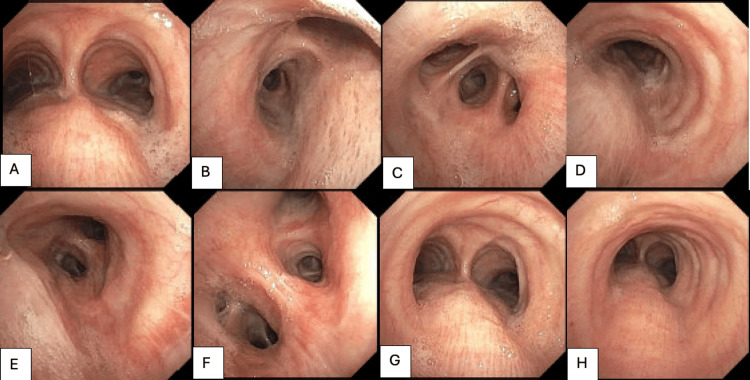
Bronchoscopy post-three months of systemic chemotherapy showing the resolution of mucosal lesions along the tracheobronchial tree in the trachea (A), right mainstem bronchus (B), right bronchus intermedius (C), left mainstem bronchus (D, E, F), and trachea (G, H).

Per the ID specialists, he was started on HAART with bictegravir, emtricitabine, and tenofovir alafenamide (Biktarvy), and the oncology service was consulted for initiation of chemotherapy. The patient was started on liposomal doxorubicin every 21 days. He received his first cycle in the hospital and was subsequently transferred to the National Institutes of Health (NIH) in Bethesda, Maryland, United States, for further treatment due to the prohibitive cost. He completed a total of three cycles of chemotherapy with doxorubicin, at which point restaging via a repeat bronchoscopy was recommended. A bronchoscopy less than three months after the initial presentation showed complete resolution of the lesions as demonstrated in Figure 3, and the patient endorsed the resolution of hematemesis as well. In light of these findings, it was decided that his tumor burden was no longer substantial enough to warrant additional cycles of chemotherapy, and the patient was maintained on Biktarvy monotherapy. At that time, his HIV RNA viral load was <20 copies/mL with CD4 count remaining <35 uL.

## Discussion

A diagnosis of KS is often clinical, but a biopsy is required for definitive diagnosis, which demonstrates spindle cell vascular proliferation within the dermis [1]. In patients with AIDS-associated KS, the mainstay of treatment is the use of HAART to treat the underlying HIV [1,2]. Systemic chemotherapy with liposomal anthracyclines (doxorubicin, daunorubicin) or paclitaxel may also be indicated depending on the severity of systemic involvement [4]. The criteria for treatment with chemotherapy include patients with >25 skin lesions, extensive oral involvement, symptomatic visceral involvement, rapid progression, and/or marked edema [1-3]. 

An endoscopic biopsy has been described as the gold standard for diagnosis in patients with concerning lesions of the GI tract [5]. However, this was not possible for our patient due to his recent DAPT use. In cases where confirmatory diagnostic testing is contraindicated and the patient has known localized KS with symptoms concerning for visceral spread, empiric treatment has resulted in the resolution of visceral symptoms [6]. Alternatively, a bronchoscopy may be another option for diagnosis, as described in our patient. Additional diagnostic challenges faced by other providers may be that nonspecific symptoms in patients without known KS do not immediately lead to a diagnosis of visceral KS being high on the differential [7]. Patients with visceral KS rarely may not have cutaneous manifestations, which further decreases the index of suspicion for this disease process and may lead to diagnostic delays [8].

This patient was uninsured and, unfortunately, unable to afford chemotherapy. However, he was eligible to enroll in a program through the NIH, which provided treatment at no cost. This program is an excellent option for patients with disseminated KS presenting to healthcare centers without local expertise in management and/or having significant financial barriers.

## Conclusions

This case demonstrates a rare case of disseminated AIDS-related KS with uncommon pulmonary involvement. It also calls attention to the utility of bronchoscopy as an alternate means of diagnosis of visceral KS in patients with suspected GI involvement and contraindications to endoscopy. Most importantly, it highlights resources available through the NIH for free treatment options for KS patients with financial constraints. This patient had an advanced case of KS with a poor prognosis but was able to receive free treatment. This led to the resolution of his symptoms and tracheobronchial lesions. Close follow-up and medication adherence will be necessary to maintain remission of his visceral disease. In the future, the index of suspicion for visceral KS should remain high in AIDS patients with nonspecific symptoms, flexibility in diagnostic methodologies should be used, and the NIH program should be heavily utilized, especially in low-resource settings.
